# Comparative analysis of esophageal gland microbes between two body sizes of *Gigantopelta aegis*, a hydrothermal snail from the Southwest Indian Ridge

**DOI:** 10.1128/spectrum.02959-24

**Published:** 2025-02-24

**Authors:** Anning Mou, Xinlong Li, Zhong Li, Lingyun Qu, Yue Dong, Zongling Wang, Xuelei Zhang, Qinzeng Xu

**Affiliations:** 1 Key Laboratory of Marine Eco-Environmental Science and Technology, First Institute of Oceanography, Ministry of Natural Resources, Qingdao, China; 2 Laboratory for Marine Ecology and Environmental Science, Qingdao Marine Science and Technology Center, Qingdao, China; 3 College of Environmental Science and Engineering, Ocean University of China, Qingdao, China; 4 National Engineering Laboratory for Integrated Aero-Space-Ground-Ocean Big Data Application Technology, Xi'an, China; 5 Qingdao Marine Engineering Survey, Design and Research Institute Co., Ltd., Qingdao, China; University of Minnesota Twin Cities, St. Paul, Minnesota, USA

**Keywords:** deep-sea hydrothermal zone, Southwest Indian Ridge, esophageal gland microbiota, *Gigantopelta aegis*, body size

## Abstract

Microbial communities within animals provide nutritional foundation and energy supply for the hydrothermal ecosystem. The peltospirid snail *Gigantopelta aegis* forms large aggregation in the Longqi vent field on the Southwest Indian Ridge. This endemic species is characterized by a changeable diet and morphology, especially reflected in internal organs such as remarkably enlarged esophageal glands. Here, 16S full-length rRNA gene analysis was performed to compare the variations in esophageal gland microbiota between two body size groups (small and large) of *G. aegis*. Phyla Proteobacteria and Bacteroidetes were the dominant featured bacteria contributing to the microbial community. No significant differences between the small and large groups were revealed by the diversity index and principal component analysis (PCA) clustering. The differences were in the relative abundance of bacteria. Compared with small-sized snails, the larger ones housed more *Thiogranum* (9.94% to 34.86%) and fewer *Sediminibacterium* (29.38% to 4.54%). Functional prediction for all of the microbiota showed that the pathways related to metabolism appeared highly abundant in smaller *G. aegis*. However, for the larger ones, the most distinctive pathways were those of environmental information processing. Facultative symbiotic *Sulfurovum* was marked as a core node in the co-occurrence network and suggested an influence on habitat selection of *G. aegis* in hydrothermal fields. In summary, variations in bacteria composition and potential functions possibly reflected changes in the anatomical structure and dietary habits of *G. aegis*. These dominant bacteria shared capabilities in nutritional supplementation and ecological niche expansion in the host, potentially a key adaptation for hydrothermal survival.

**IMPORTANCE:**

Dominant in the Longqi hydrothermal vent Southwest Indian Ridge, *Gigantopelta aegis* was observed to undergo unique and significant morphological changes and diet shifts known as cryptometamorphosis. During this process, *G. aegis* developed a specialized bacteria-housing organ, the esophageal gland, in the later life stages. Our research discovered variations in esophageal gland microbes between different body size groups of snails. These bacteria were closely related to the development and health of *G. aegis*. Full-length 16S rRNA gene analysis revealed more *Thiogranum* and fewer *Sediminibacterium*, suggesting a potential association with environmental adaptation. In the small-sized group, the potential functions were enriched in metabolism, while in larger *G. aegis* individuals, predictions indicated adaptive functions such as environmental information processing. Also, symbiotic *Sulfurovum* could be one of the factors influencing the habitat selection of *G. aegis*. Understanding the complex relationship between benthic macrofauna and microbes helps us describe the mechanisms of survival in extreme environments.

## INTRODUCTION

Deep-sea hydrothermal vents are characterized by high temperatures, intense pressure, and darkness, and are rich in heavy metals released from hydrothermal alteration (1, 2). Despite those extreme conditions, a unique set of organisms resides there (3), including giant tubeworms, mussels, clams, eyeless shrimp, crabs, and snails (4). All these benthic macrofauna have associated microbes in their tissues or specialized organs, such as the trophosome, gills, external surfaces, and gastrointestinal tract (5). The functional traits of microbes in above tissues are especially important (6). As an illustration, the sulfur-oxidizing endosymbionts in the trophosomal tissue can provide both energy and organic carbon for hosts (7). In *Munidopsis alvisca* and *Shinkaia crosnieri*, epibionts serve as a nutrient source and aid in sulfide detoxification (8, 9).

The gastrointestinal microbiota is contributable to the adaptation and health of various deep-sea benthic macrofauna under vent fluid chemistry conduction (10–13). Many studies have revealed that the resident gut microbiota of *Deferribacterota* produced essential compounds from minerals ingested by the shrimp *Rimicaris exoculata* (14, 15). The functional traits of gastropod microbiomes have attracted considerable research interest (16). Gut microbial ecology supports the nutritional and metabolic demands of snails, as demonstrated in *Alviniconcha marisindica* (17). γ-Proteobacteria in the esophageal gland of scaly-foot gastropods (*Chrysomallon squamiferum*) potentially export proteins from the bacterium to the host (18). Given the important roles of gastrointestinal microbes, the factors influencing their community structure have prompted extensive exploration (19). The influence of life cycle and morphological changes on microbial composition has been confirmed in *Rimicaris* spp. (20–23). In another instance, correlative imaging analyses concluded that the body plan is an important driving factor for microbial variation in bathymodioline mussels (*Bathymodiolus* sp.) (24, 25). Many studies have been conducted on deep-sea hydrothermal benthic macrofauna; however, factors contributing to changes in the gastrointestinal microbiota of gastropod mollusks, a dominant taxon (26), remain scarce.

The gastropod taxon in hydrothermal vents exhibits a rich species diversity. The snails *Alviniconcha* (27), *Gigantopelta* (28), *Chrysomallon* (29), and the limpet *Lepetodrilus* (30) all belong to this taxonomic group. Among the categories, the genus *Gigantopelta* features a unique “cryptometamorphosis” phenomenon in its two species: *Gigantopelta chessoia* and *Gigantopelta aegis* (31). They have the ability to feed via radular grazing in early life stages. As they grow in size, these snails markedly enlarge and rapidly shift to relying on chemosynthetic microbes for essential sustenance (28). *Gigantopelta aegis* (Neomphalida: Peltospiridae) was one of the endemic species with a sulfide coating found at the Longqi vent on the Southwest Indian Ridge (SWIR) (28, 32, 33). The significantly enlarged esophageal glands provide space for bacteria. Due to the changes in morphology and feeding mentioned above, *G. aegis* snails serve as a valuable model for studying the impact of size variation on gastrointestinal microbes composition. Hologenome analysis revealed the adaptation of *G. aegis* to deep-sea hydrothermal vents and the dual symbiosis between two γ-proteobacterial microbes (34, 35). The composition and function of esophageal gland microbes in *G. aegis* across different body sizes remain unclear (36).

In previous researches, we observed dense aggregations of the deep-sea snail *G. aegis* in the hydrothermal field of the SWIR. In this study, we adopted full-length 16S rRNA gene to analyze the esophageal gland microbes of *G. aegis* at different body sizes to (i) uncover the diversity and compositional structure of microbial communities, and (ii) predict the microbe functions that facilitate the environmental adaptation of *G. aegis*.

## MATERIALS AND METHODS

### Deep-sea sampling and identification

**Fig 1:**
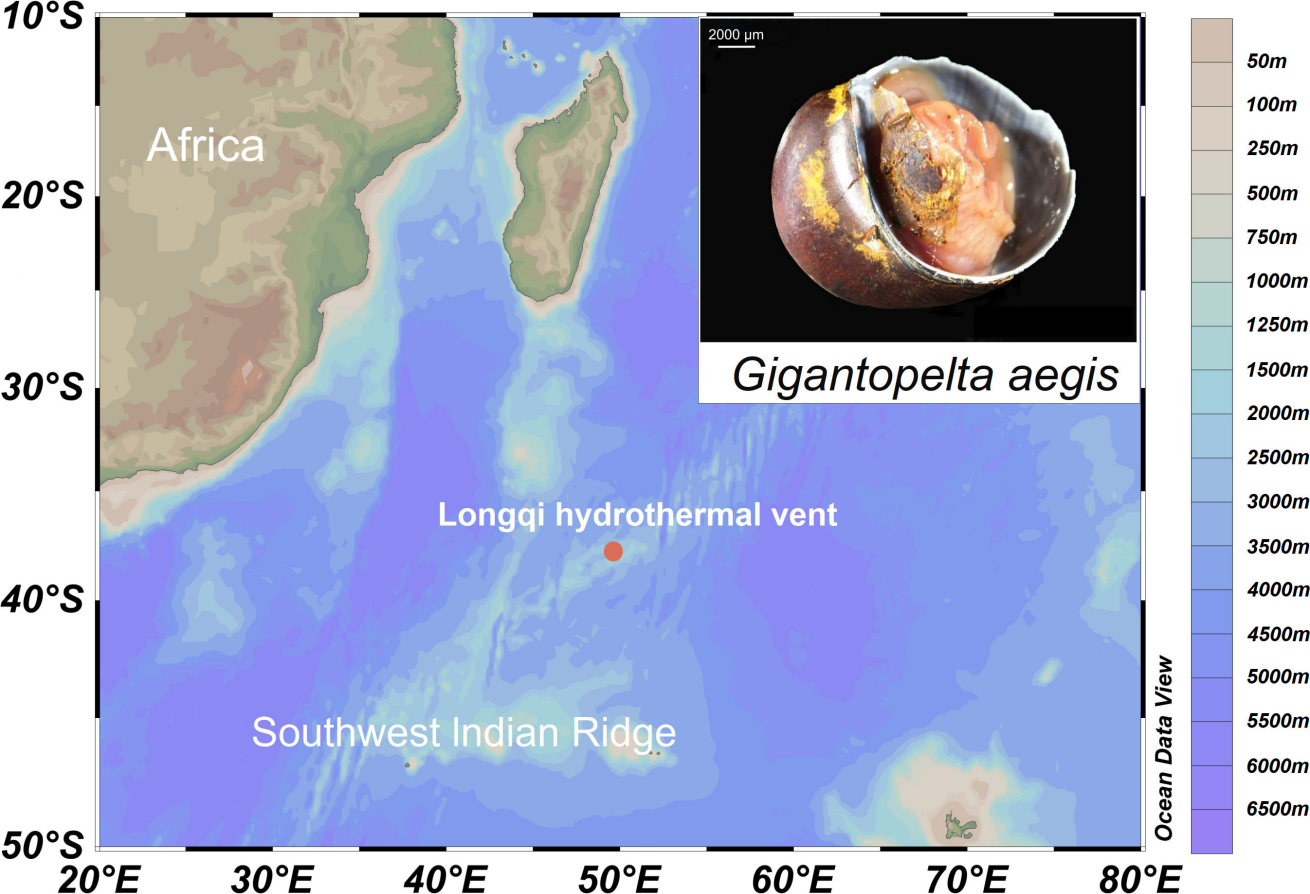
Sampling site at the Longqi hydrothermal vent field in the Southwest Indian Ridge.The sampling site map was created using the software package Ocean Data View v.5.5.2.

*Gigantopelta aegis* snails were collected using remotely operated vehicle (ROV) Hailong Sanhao at a hydrothermal vent on the SWIR (37.78°S, 49.65°E; 2,755 m; Fig. 1) during the third leg of the Dayang Yihao oceanographic expedition DY52 in April 2019. Once onboard, specimens were preliminarily identified by morphology and immediately stored in 75% ethanol at −20°C or frozen at −80°C. After returning to Qingdao, all snails were transported to the laboratory at the First Institute of Oceanography, Ministry of Natural Resources. We dissected 10 mg foot muscle tissue and extracted DNA using the E.Z.N.A. MicroElute Genomic DNA kit (Omega Bio-tek, Norcross, GA, USA) for subsequent PCR. Detailed primer information is provided in Tables S1 and S2 (37–39). Unpurified PCR samples were sent to Sangon Biotech (Shanghai) Co., Ltd. for sequencing. The sequencing results were verified using BLAST (with >99% identity) in the National Center for Biotechnology Information database, and we finally identified the biological samples as *G. aegis*.

To avoid contamination, we selected 12 intact snail individuals from all identified *G. aegis*. We measured the shell length from the top to the aperture and divided the individuals into two groups: the small group (6 individuals, 5 mm–10 mm, 3 pooled samples) and the large group (6 individuals, 17 mm–40 mm, 6 independent samples). The length of individuals<10 mm have only minimal esophageal gland tissue (28), and the DNA extracted from a single sample is insufficient for downstream sequencing. Following the same approach used in *G. chessoia* (40), we performed pooling. Each sample in the small group was composed of two similarly sized individuals (Table S2). The shells were removed under a stereomicroscope (Nikon SMZ270, Japan), and esophageal gland tissues were obtained for microbial DNA extraction.

### Microbial DNA extraction and sequencing

Microbial DNA was extracted from the esophageal gland of *G. aegis* using the E.Z.N.A. Soil DNA kit (Omega Bio-tek, Norcross, GA, USA) according to manufacturer’s instructions. The V1-V9 region of the bacterial 16S rRNA gene was amplified using primers 27F: 5′-AGRGTTYGATYMTGGCTCAG-3′ and 1492R: 5′-RGYTACCTTGTTACGACTT-3′ (41). Amplifications were performed with the following PCR program: 95°C for 2 min, followed by 27 cycles at 95°C for 30 s, 55°C for 30 s, and 72°C for 60 s, and a final extension at 72°C for 5 min. PCR reactions were carried out in triplicate, and each sample was amplified in a 20 µL reaction mixture containing 4 µL of 5 × FastPfu Buffer, 2 µL of 2.5 mM deoxynucleotide triphosphates (dNTPs), 0.8 µL of each primer (5 µM), 0.4 µL of FastPfu Polymerase, 10 ng of template DNA, and make up to 20 µL with ddH_2_O. Amplicons were extracted from 2% agarose gels and purified using the AxyPrep DNA Gel Extraction Kit (Axygen Biosciences, Union City, CA, USA). Based on preliminary electrophoresis results, the PCR products were quantified by QuantiFluor ST blue fluorescence quantitation system (Promega Corporation).

The amplified DNA products were then used to prepare SMRTbell libraries by blunt-ligation (Pacific Biosciences). Purified SMRTbell libraries from the Zymo and HMP mock communities were sequenced on dedicated PacBio Sequel II 8M cells using the Sequencing Kit 2.0 chemistry. Additionally, purified SMRTbell libraries from the pooled and barcoded samples were sequenced on a single PacBio Sequel II cell. Amplicon sequencing was performed by Shanghai Biozeron Biotechnology Co., Ltd (Shanghai, China). PacBio raw reads were processed using the SMRT Link Analysis software (version 9.0) (42) to obtain demultiplexed circular consensus sequence reads with the following settings: minimum number of passes = 3, minimum predicted accuracy = 0.99. Raw reads were processed through SMRT Portal to filter sequences for length (<800 or >2,500 bp) and low quality.

### Data filtering and taxonomic classification

Sequences were further filtered by removing barcodes, primer sequences, chimeras, and sequences if they contained 10 consecutive identical bases. Operational taxonomic units (OTUs) were clustered with 98.65% similarity cutoff using UPARSE (version 7) (43), and chimeric sequences were identified and removed using UCHIME (44). The phylogenetic affiliation of each sequence was analyzed by RDP Classifier against the Silva SSU rRNA database (version 138) (45) using confidence threshold of 80%. The final OTU table included only sequences classified within bacterial phyla.

### Diversity and statistical analysis

Alpha-diversity and evolutionary relationships were calculated for diversity metrics based on Chao1 index, Shannon index, Pielou’s index, and phylogenetic diversity (PD) index. The *t*-tests and standard error were used to detect the differences between groups in R 4.3.1. Principal component analysis (PCA) was applied to log2-transformed data (46) to reflect the difference and distance between samples at the OTU level as well. We performed analysis of similarity (ANOSIM) to evaluate the differences in the microbiota communities (47). Similarity percentage analyses were used to identify the contribution of each taxon to the community dissimilarity (47). These two analyses were conducted with vegan v.2.6-4 package (48) in R. To assess the significance of differences of biomarkers in two *G. aegis* groups, the linear discriminant analysis (LDA) effect size (LEfSe) analysis was carried out (49). We used a log10 transformation, and identified biomarkers with LDA scores >4 through the microeco v.1.6.0 package (50).

### Analysis of potential functions

The potential functions of bacterial communities based on 16S rRNA gene sequences were annotated using the Phylogenetic Investigation of Communities by Reconstruction of Unobserved States (PICRUSt 2.0) v.2.5.2 (51) with Kyoto Encyclopedia of Genes and Genomes (KEGG) Orthology (KO) (52). The abundances of predicted functional pathways were normalized to sequencing depth and expressed as percentages of the total number of predicted functions from the KO database. Pairwise comparison analysis was performed for the bacterial function pathways of *G. aegis* microbial communities, including the morphologically small-sized group and large-sized group. The Statistical Analysis of Metagenomic Profiles v.2.1.3 analysis indicated significant variations in pathways at KEGG level 2 (53), using Welch’s *t*-test with default parameters. The scatter fit curve reflected the status of those differential pathways in our samples using Origin 2021 software.

### Phylogenic and co-occurrence network analysis

To investigate any potential symbionts among the dominant bacteria, the OTUs of the *G. aegis* esophageal gland microbiota were subjected to BLASTn searches against the GenBank database with a threshold of identity >90.00%. Among OTUs, the genus *Sulfurovum* showed homology to known hydrothermal field symbionts. To further elucidate the microbial candidates, 10 OTUs of *Sulfurovum*, their top BLAST hit sequences (Per.Ident >95%), and 16S rRNA bacterial sequences from different genera within the same family were aligned using ClustalW and manually trimmed. Neighbor-joining (NJ) methods was selected for phylogenetic analysis using MEGA (version 11) (54). The best model of Kimura two-parameter (K2) combined with a gamma distribution (G) and a proportion of invariable sites (I) was selected by the model test in MEGA, and 1,000 bootstrap replications were used. Three *Methylomarinum vadi* OTUs and the 10 most abundant γ-proteobacterial OTUs were respectively clustered with their top 3 BLAST hit sequences and the primary *G. aegis* symbionts that had been previously identified (34). The phylogenetic tree used to calculate the PD index at the OTU level was also obtained through MEGA (version 11) with the aforementioned model.

A co-occurrence network analysis was calculated using R igraph v.2.0.3 and *Hmisc* v.5.1-3 packages (55, 56). Data with a discovery rate below 20% were filtered out. Spearman’s correlation coefficients and Benjamini-Hochberg false discovery rate method (57) were used for multiple testing correction (*|r|* > 0.6; *P* < 0.001). The visualization was implemented in Gephi software (version 0.10.1) (58).

## RESULTS

### Structural characteristics of the microbial communities

After quality filtering, raw sequences from the esophageal gland of *G. aegis* generated 106,369 high-quality reads, with 99.16% of sequences being 1,301 bp–1,500 bp in length, ranging from 9,796 to 17,442 reads per sample. All the clean reads were clustered into 4,863 OTUs and included in downstream analyses. The rarefactions for the nine studied samples were saturated at 5,377 reads per sample, indicating plateau (Fig. S1). There were 732 OTUs common to both groups, with 3,189 unique OTUs in the large group and 942 in the small group. However, over 90% of the unique OTUs in the large group were clustered with no more than five sequences.

**Fig 2:**
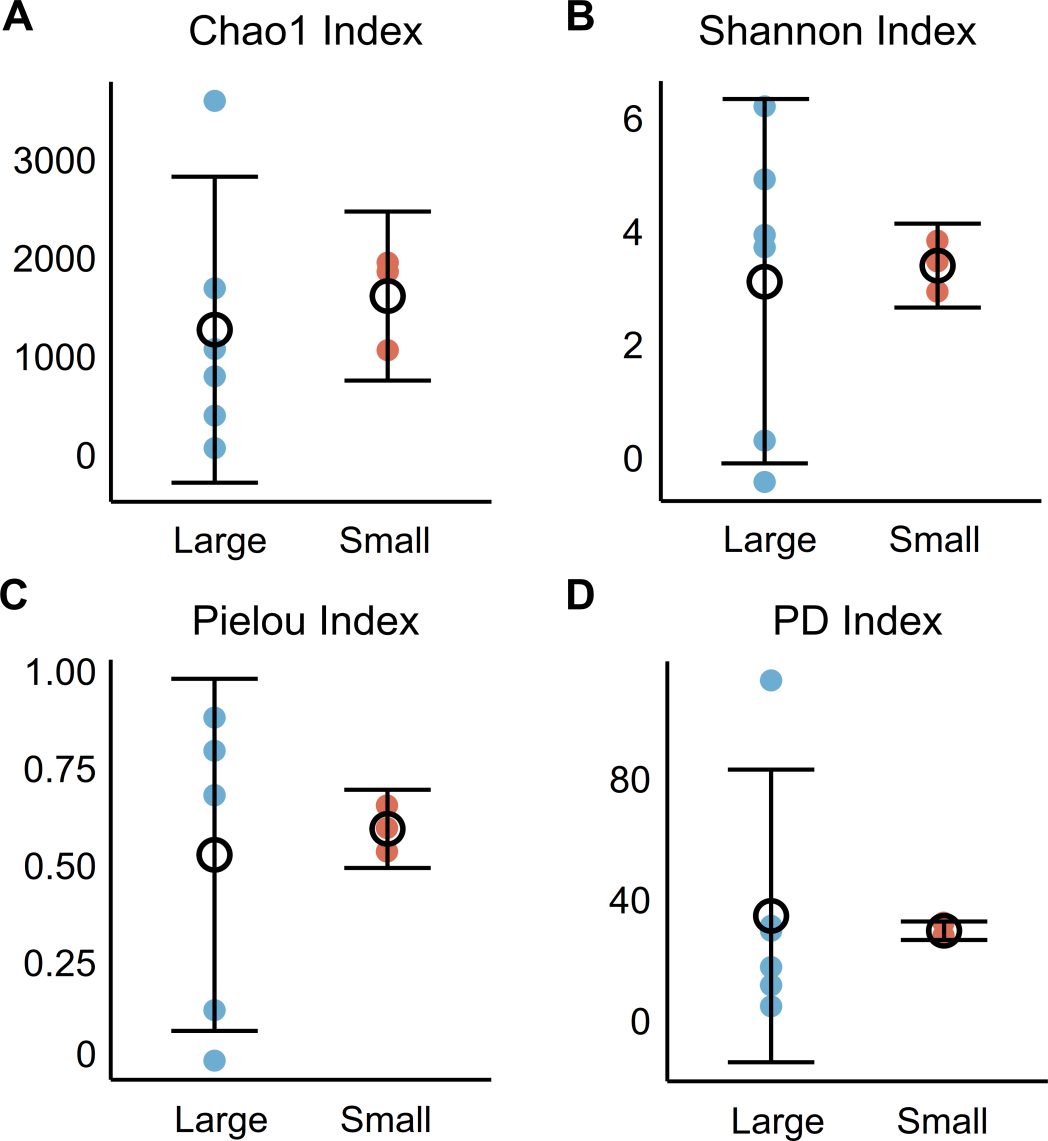
OTU alpha-diversity analyses of the *Gigantopelta aegis* esophageal gland microbiota. Alpha-diversity was calculated using Chao1 index, Shannon index, Pielou index, and PD index, and the trend of data distribution was illustrated using error point plots. Statistical analysis was conducted on alpha-diversity using *t*-tests. NS, not significant; *P* > 0.05.

**Fig 3:**
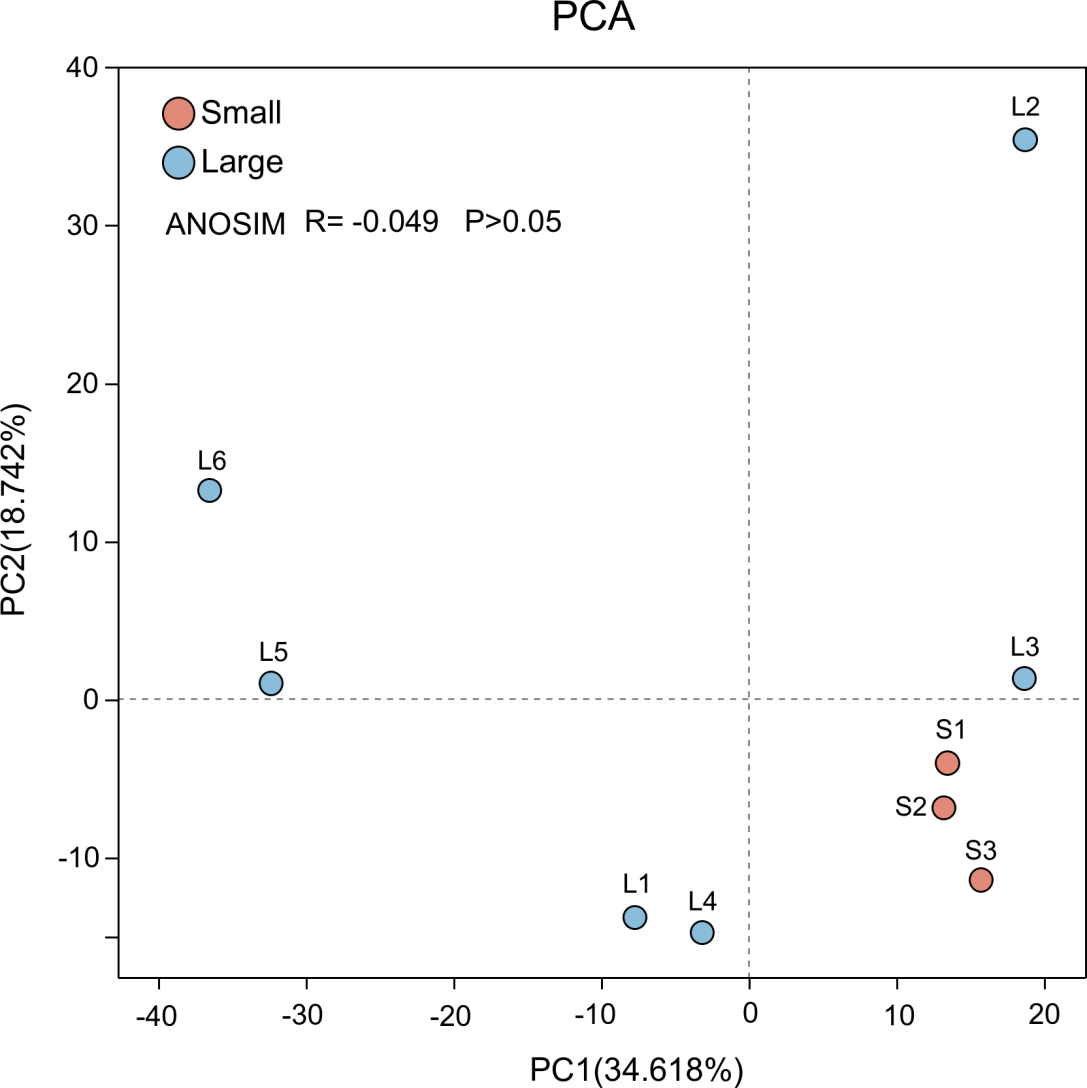
PCA plot of esophageal gland microbiota in *Gigantopelta aegis* under OTU level. *R* = −0.049, *P* > 0.05; No significant differences between groups, but existing difference within the large.

In terms of alpha-diversity (Fig. 2), richness, diversity, evenness, and phylogenetic distance were compared between two groups. The Chao1 index and PD index points in large-sized group were generally lower than those in the small-sized group (*P* > 0.05). The results for the Shannon index and Pielou index were the opposite (*P* > 0.05). Based on Bray-Curtis distance, principal component analysis revealed the discrete clustering of *G. aegis* microbiomes (Fig. 3). The first two axes of PCA accounted for 53.36% of the variation, and the microbiota of small *G. aegis* were not separated from the large *G. aegis* community. The difference of microbiomes between two groups were also not statistically significant based on the results of ANOSIM (*R* = −0.049, *P* > 0.05). Overall, the results of alpha-diversity and beta-diversity did not demonstrate the independence of esophageal gland microbiota between small and large *G. aegis* snails.

### Compositions of the microbial communities

**Fig 4:**
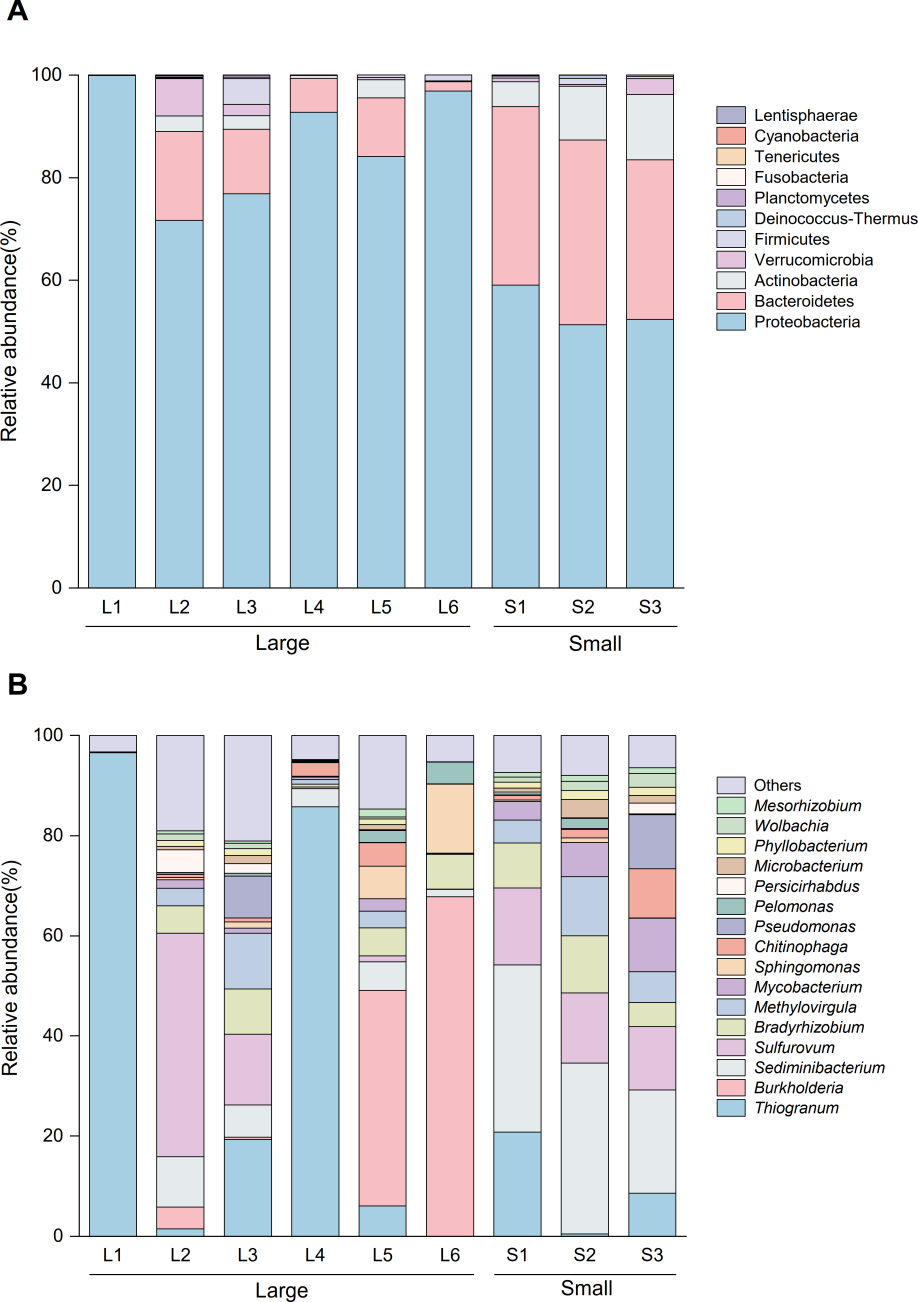
Taxonomic composition and similarity of gut microbiota at the phylum level (**A**) and genera level (**B**) in *Gigantopelta aegis* from the hydrothermal region of Longqi, SWIR. Others, the taxonomic groups with abundances less than 1% at the genus level.

The annotated esophageal gland microbiota of *G. aegis* included 11 phyla and 255 genera. At the phylum level (Fig. 4A; Table S3), Proteobacteria was the predominant microbiota in both groups, accounting for 54.06% in the small group and 85.96% in the large group. The phyla Bacteroidetes (33.91% and 9.34%) and Actinobacteria (9.34% and 1.65%) also had relatively high abundance in the two body sizes of snails, with varying abundance between the two groups. Significant differences have been observed in the two groups of bacterial communities at the phylum level, as indicated by ANOSIM (*R* = 0.926, *P* < 0.05). Tenericutes only appeared in the large group, while Lentisphaerae was unique to the small group.

Among all identified genera (Fig. 4B), more detailed divergences were found in the two groups (*R* = 0.012, *P* > 0.05). *Sediminibacterium* was the most dominant group in small groups (29.38%), while only 4.54% were detected in another group. The genus *Sediminibacterium* significantly contributed to the differences between the two groups (*P* = 0.001, Table S4). The genus *Thiogranum* was commonly found in collected *G. aegis*, with varying abundance: 34.86% in the large group and 9.94% in the small group. The genus had a similar abundance in *Sulfurovum* (13.99% of small group and 10.06% of large group). It was worth noting that the abundance of *Burkholderia* in large-sized snails (19.28%) was much higher than in small-sized snails (0.01%).

**Fig 5:**
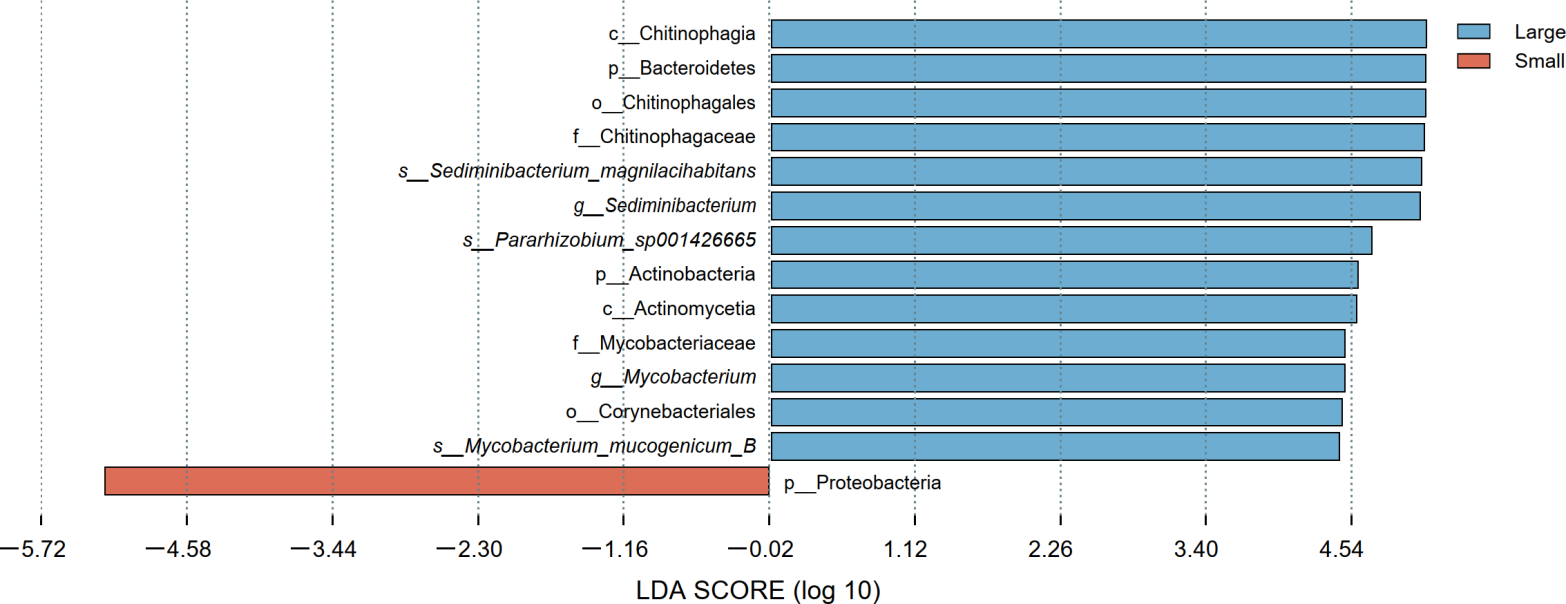
Characterization of esophageal gland microbiota in *Gigantopelta aegis* by LEfSe and a histogram of the LDA scores (log10). s, species; g, the genus; f, the family; o, the order; c, the class; p, the phylum.

Moreover, the bacterial biomarkers of the two groups showed significant differences at various taxonomic levels (Fig. 5). The LDA scores indicated that the phylum Proteobacteria was the largest contributor to the intergroup differences in the small group; the class Chitinophagia had a significant contribution to the large group. Additionally, the phylum Actinobacteria, particularly the genus *Mycobacterium* (*P* = 0.001, Table S4), was also an important contributor to the differences in the large hydrothermal *G. aegis*.

### Potential functions of esophageal gland microbiota

In level 2 KOs, a total of 34 pathways were predicted by PICRUSt analysis, which belonged to five categories: metabolism, genetic information processing, cellular processes, organismal systems, and environmental information processing.

**Fig 6:**
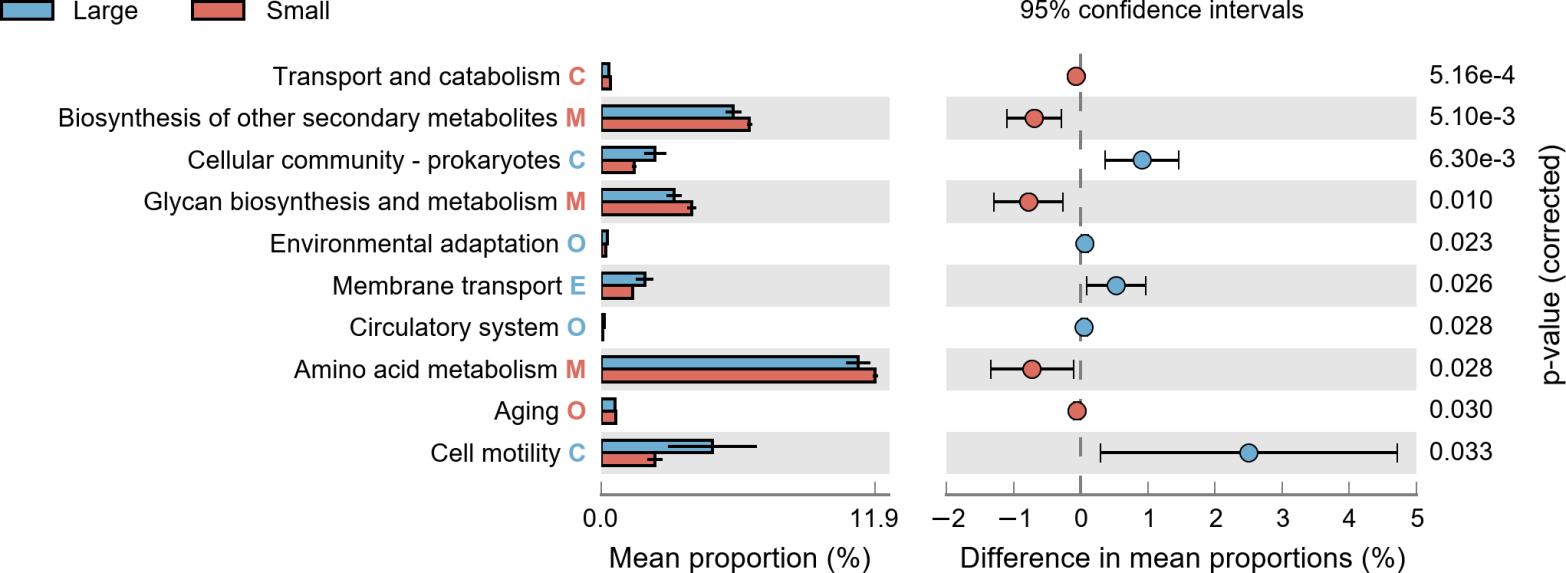
Significant KEGG pathways in esophageal gland microbiota of *Gigantopelta aegis*. Significantly expressed pathways at KEGG level 2 in the gut microbiota of *G. aegis* based on PICRUSt2 analysis (*P* < 0.05). C, cellular processes; E, environmental information processing; M, metabolism; O, organismal systems.

The significant divergence in 10 potential KEGG pathways was found between the small and large *G. aegis* (*P* < 0.05, Fig. 6). Based on the predicted results, it is speculated that in the small group, the primarily enriched KOs include biosynthesis of other secondary metabolites, glycan biosynthesis and metabolism, and amino acid metabolism, which belonged to metabolism pathways. The large group, on the other hand, tends to be enriched in pathways such as cellular community—prokaryotes, environmental adaptation, membrane transport, circulatory system, and cell motility. Analysis results speculated a remarkable enrichment of pathways under environmental information processing in larger *G. aegis* (Fig. S2).

### The phylogenetics of mainly *Sulfurovum*, the symbiotic bacteria in *G. aegis*

**Fig 7:**
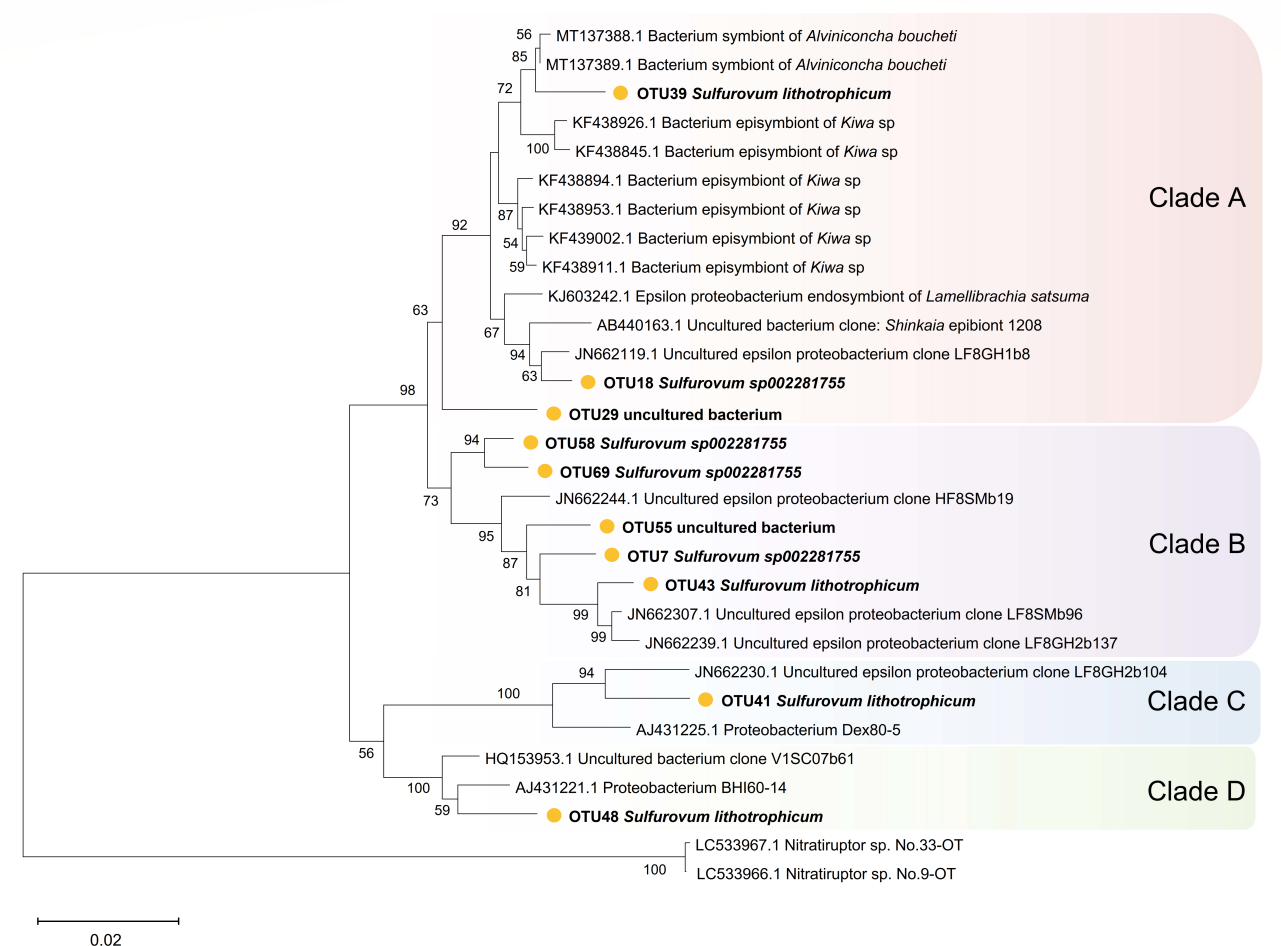
Phylogenetic analysis of *Sulfurovum*. The bootstrap values (>50%) of relevant nodes are shown based on 1,000 replicates. The number on the branch was the result of the NJ algorithm. Sequences from this study are shown in bold.

Phylogenetic analysis indicated that OTU39 of the genus *Sulfurovum* from *G. aegis* was clustered with bacterial symbionts of some other hydrothermal animals in clade A (Fig. 7). Those bacteria included symbionts of the snail *Alviniconcha boucheti* (16), episymbionts of *Kiwa* sp. (59), endosymbionts of *Lamellibrachia satsuma* (60), and epibionts of *Shinkaia crosnieri* (61). In PICRUSt annotation, OTU39 is frequently mentioned in pathways related to amino acid metabolism, biosynthesis of other secondary metabolites, and glycan biosynthesis and metabolism. Clade B comprises five OTUs nested among three uncultured epsilon proteobacteria collected from tubeworm (*Ridgeia piscesae*) aggregations (62). In clades C and D, our *Sulfurovum* OTU primarily clustered with bacteria from deep-sea vent *Alvinella* spp. worm habitats (63), along with some proteobacteria in microbial mats from various hydrothermally active environments (64).

In addition to *Sulfurovum*, other bacteria closely related to the known symbionts of *G. aegis* were detected, including the sulfur-oxidizing *Thiogranum* and the methane-oxidizing *M. vadi,* as shown in Fig. S3 and S4.

### Microbial co-occurrence network

**Fig 8:**
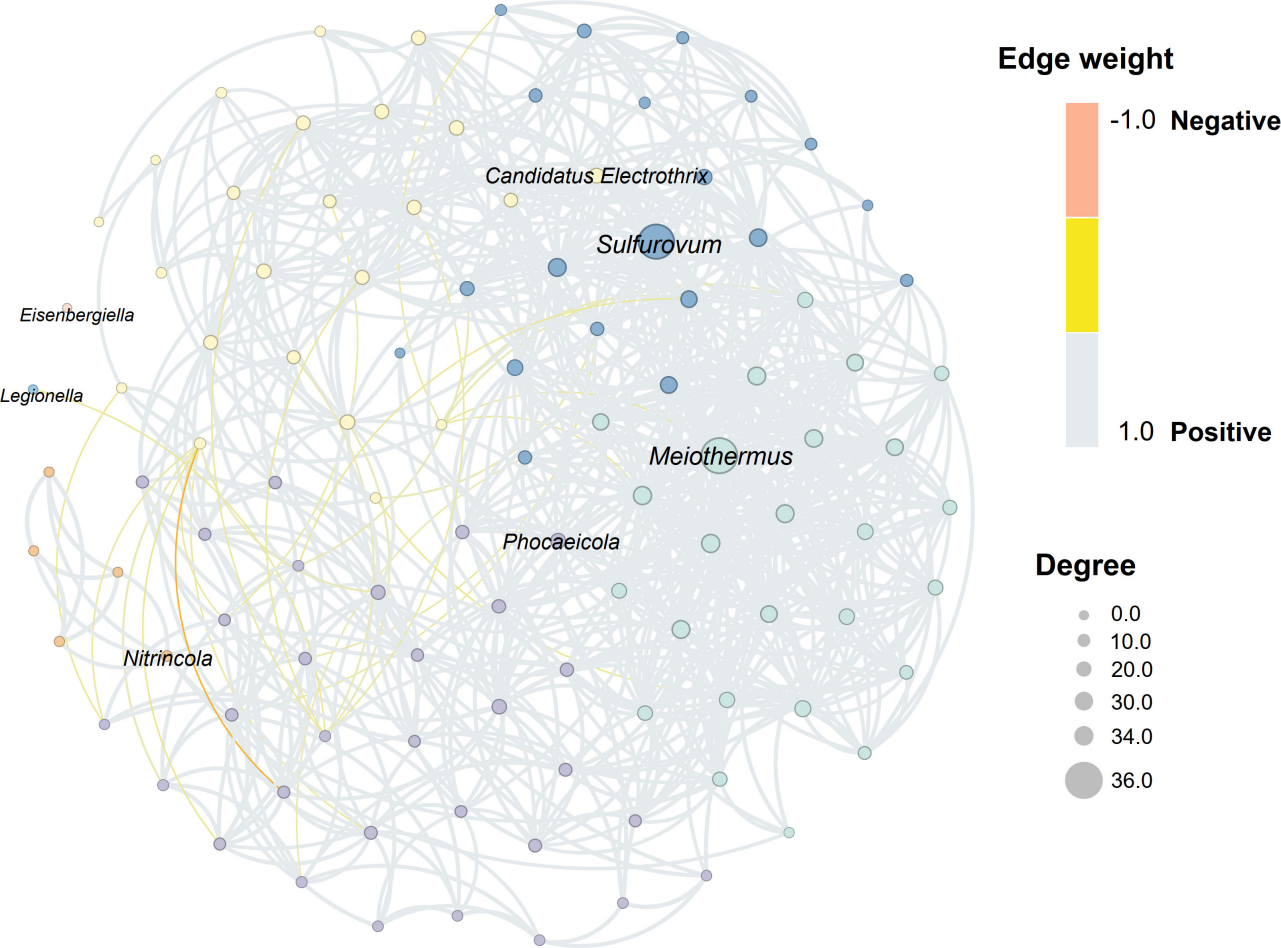
Microbes with a detection rate of >20% are used to construct the co-occurrence network. Color of nodes indicates genus from the same module in network, lines represent positive or negative correlations between nodes. Spearman’s correlation coefficient; *r* < −0.6 or *r* > 0.6, *P* < 0.001.

Constructed with 106 microbial nodes and 830 edges (Fig. 8), the co-occurrence network reflected strong microbial interactions by the average clustering coefficient (0.583) and path length (2.447), representing the complexity and compactness of the network property. The average weighted degree (15.66) with modularity (0.426) revealed the strong connections among nodes. Overall, positive correlations were the main feature of the bacterial community. *Meiothermus*, *Sulfurovum*, *Candidatus Electrothrix*, *Phocaeicola*, and *Nitrincola* were the largest nodes in each module (degree: 36.0, 35.0, 23.0, 23.0, and 7.0). *Legionella* and *Eisenbergiella* had only few connections with other microbes, forming isolated modules (degree: 1.0 and 0.0). *Thiogranum* and *Pelomonas* showed the strongest negative correlation among all microorganisms.

## DISCUSSION

### Esophageal gland bacteria affected by body size of *G. aegis*

Previous anatomical studies have observed a specialized bacteria-housing organ developed in the later stage of post-settlement life in *Gigantopelta*, which was derived from the esophageal gland (28, 31). This specialized organ was similar to the light organ developed in adult ceratioid anglerfish (*Cryptopsaras couesii*), capable of holding bioluminescent bacteria (65). Differences in bacterial composition have been revealed between the larvae and adults of *C. couesii* because of the light organ. Studies have shown that gut microbiota communities had different structures and function tendency depending on the changes of animal body size (20). Our results also demonstrated the impact of body size on esophageal gland microbes of *G. aegis*. Proteobacteria and Bacteroidetes were the predominant microbial phyla in two body size groups, respectively (Fig. 4). These bacteria have been found in a variety of deep-sea mollusk tissues, such as the gills of limpet (*Lepetodrilus fucensis*) (66) and mussels (*Gigantidas haimaensis*) (10). Moreover, the genus *Sediminibacterium* (Bacteroidetes) exhibited significant differences between the two groups (small: 29.38%, large: 4.45%; *P* = 0.001; Table S4). There were also differences of γ-proteobacterium *Thiogranum* (9.94%; 34.86%) and β-proteobacterium *Burkholderia* (0.01%; 19.28%) between two body size groups. Xiao et al. (10) reported that more γ-proteobacteria and fewer Bacteroidetes suggested increased methane use and loss of dietary polysaccharides. Our study also observed such changes in bacteria abundance, and we consider this potentially related to the *Gigantopelta* digestive system degradation and diet shifts (67).

Many metabolic pathways exhibited differences between small and large *G. aegis* according to the PICRUSt results (Fig. 7). The predicted results indicate that the microbiota of small individuals includes more biosynthesis and metabolic functions, while the esophageal gland microbiota of the large group is more related to environmental adaptation and communication. As revealed in deep-sea hydrothermal vent benthic macrofauna, the dominant microbes were the main contributors to the host metabolism (68). The bacteria *Sediminibacterium* was mainly engaged in biosynthesis and degradation processes, such as polysaccharide and amino acid metabolism, in acidic and thermophilic environments (69). *Sediminibacterium magnilacihabitans* had the highest OTU count (84.44%) in its genus. It possessed the capability for complex carbon degradation (such as cellulose and chitin) and nitrate reduction (70, 71). The metabolic products could be converted into small molecular weight carbohydrates and nitrogen-containing compounds while releasing energy (72). These bacterial metabolic processes contribute to material synthesis in small snails. Having increased by over threefold in large snails, SOX *Thiogranum* was capable of facilitating metal-sulfide dissolution via extracellular electron transfer (73). It also participated in nitrogen fixation and denitrification alongside *Bradyrhizobium* (74). Furthermore, *Thiogranum* and *M. vadi*, two potential symbionts (Fig. S3 and S4), may form a dual symbiotic system that is highly versatile in utilizing chemical substances such as methane, hydrogen, sulfides, and nitrogen-containing compounds (34). *Burkholderia* was another bacterium abundant in large *G. aegis*, previously found in hydrothermal sediments and the guts of surrounding animals (75–77), characterized by high metal tolerance and efficient consumption of thiosulfate and nitrite (78–80). The increase in *Thiogranum* and *Burkholderia* may be a result of adaptation to the abundant sulfide deposits in the SWIR hydrothermal field (81). The phylum Tenericutes (Haloplasma), not a dominant bacterium and found only in the small group, was previously identified by Ku et al. (82) as having proteins related to cell motility. However, due to the challenges in deep-sea sampling, the collected sample of *G. aegis* individuals did not comprehensively represent all growth stages. Further study is essential to integrate more biological and sediment data for refining the accuracy of our conclusions.

### Core microbiome contributes to the *G. aegis* assemblage

In our study, the *Sulfurovum* maintained a relatively stable abundance in the two body size stages of *G. aegis* (13.99%; 10.06%) and was identified as a core microbiome component (83). Phylogenetic analysis (Fig. 8) revealed that OTU39 *Sulfurovum lithotrophicum* is the closest to other symbionts. Particularly, the *Sulfurovum* species identified in *G. aegis* were homologous to those endosymbiont *Sulfurovum* found in the hydrothermal snail *Alviniconcha boucheti* (16). *Sulfurovum* are classified to be microaerophilic sulfur-oxidizing bacterium that favor to occur in high-temperature habitats (84). It frequently uses inorganic sulfides as electron donors for carbon fixation and plays a crucial role in heavy metal detoxification through metabolic activities (17, 85, 86). Previous studies have revealed that it can efficiently fix CO_2_ under high pressure through the reductive tricarboxylic acid cycle, primarily generating amino acid metabolites (67, 87). The chemoautotrophic ability of *Sulfurovum* enables it to fulfill the functions required as an important symbiont for vent animals (88). In the co-occurrence network (Fig. 6), the microbial nodes strongly correlated with *Sulfurovum* have functions in metal migration, sulfide detoxification, and redox cycles of nitrogen and carbon (89–91), contributing to the *G. aegis* environmental adaptation. For instance, *Meiothermus granaticius* can assimilate a range of organic acids and generate energy through carbon metabolism (92). The cable bacteria *Candidatus Electrothrix,* recognized as electron transporter (93, 94), are notable for their exceptional conductivity: connect sulfide oxidation with oxygen or nitrate reduction via centimeter-long filaments electron transport (95, 96).

The ε-proteobacterial *Sulfurovum* contributes to promoting biological colonization in hydrothermal ecosystems (16). For example, *Alviniconcha* holobionts, which dominate in high end-member concentrations of sulfide and hydrogen, contain ε-proteobacterial as symbionts. (97). Similarly, our sampling site at SWIR also noted for abundant sulfides (81), with hydrogen concentrations in hydrothermal fluids from the Longqi vent field being about 0.2 mmol/kg (98). Localized conditions of fluid chemistry are conducive to the survival and proliferation of *Sulfurovum* (99), and dense population of *G. aegis* (33). The species *G. aegis* has been described in previous studies as pioneers and opportunistic colonizers that appear in hydrothermal vents (100). Therefore, we have reason to speculate that the locally dominant symbiont *Sulfurovum* contributes to the community assemblage of *G. aegis*.

Additionally, almost all microbes in the esophageal gland of *G. aegis* are observed in active and inactive hydrothermal vent locations of the SWIR (101–103). A horizontal transfer model has been proposed in hydrothermal vent snails *G. chessoia* (40), and confirmed in the sister genera *Alviniconcha* and *Ifremeria* (104). Our study did not include environmental microbes or other tissues of *G. aegis*, so whether horizontal transfer of bacteria occurs in *G. aegis* still requires further experimental and analytical support.

### Conclusion

In this study, we report the esophageal gland microbes of the dominant species *G. aegis* at the SWIR Longqi hydrothermal vent, and we enhance our understanding of the contribution of microbes to the developmental processes of deep-sea benthic macrofauna. There were differences in the relative abundance of microbe categories between small and large *G. aegis*, which demonstrated a potential connection with morphological changes and diet shifts. Functional predictions suggest an enrichment of environmental adaptation pathways in larger *G. aegis*, compared to the abundant metabolic processes in the smaller group. We identified a bacterial genus that clustered closely with the symbionts found in another hydrothermal vent snail *A. boucheti*. This potential symbiont was identified as a core microbe in the esophageal gland microbiota. Overall, these results suggested an adaptation strategy for extreme environments, and provided a set of data for further research on how bacteria influence the nutritional sources and niche expansion of hydrothermal organisms.

## Data Availability

Data on esophageal gland microbes are available in NCBI SRA under BioProject accession PRJNA1162764. *Gigantopelta aegis* sequencing results of the 16S, 28S ribosomal RNA genes and COI gene are available in GenBank under accession PQ357675-PQ357686, PQ357669-PQ357674, and PQ358450-PQ358456, respectively.

## SUPPLEMENTAL MATERIAL

Supplemental figures and tables spectrum.02959-24-s0001.pdf Tables S1 to S4 and Fig. S1 to S4.

ASM does not own the copyrights to Supplemental Material that may be linked to, or accessed through, an article. The authors have granted ASM a non-exclusive, world-wide license to publish the Supplemental Material files. Please contact the corresponding author directly for reuse.
