# Supplementary material for: Comparative analysis of esophageal gland microbes between two body sizes of Gigantopelta aegis, a hydrothermal snail from the Southwest Indian Ridge
Source: Microbiol Spectr. 2025 Feb 24;13(4):e02959-24. doi: 10.1128/spectrum.02959-24 (PMC11960433; doi:10.1128/spectrum.02959-24)
Supplement: Supplemental figures and tables — Tables S1 to S4 and Fig. S1 to S4. [file spectrum.02959-24-s0001.pdf]

SUPPLEMENTAL INFORMATION

Table S1 PCR primers used for amplifying *Gigantopelta aegis*

Target Fragment	PCR Primers	Primer Sequence	PCR program
COI	LCO1490	GGT CAA CAA ATC ATA AAG ATA TTG G	95 ℃ for 15 minutes, followed by 40 cycles of 94 ℃ for 45 seconds, primer-specific annealing temperature for 60 seconds, 72 ℃ for 60 seconds, and a final extension at 72 ℃ for 5 minutes. The annealing temperatures used were 47 ℃ for 16S and 28S, and 45 ℃ for COI.
	HCO2198	TTA ACT TCA GGG TGA CCA AAA AAT CA	
28S	28SSFF	AGTAAC GGC GAG TGA AGC GGG	
	28SSFR	CGG TTT CAC GTA CTC TTG AAC TCT CTC	
16S	16Sa	CGC CTG TTT ATC AAA AAC AT	
	16Sb	CCG GTC TGA ACT CAG ATC ACG T	

Table S2 Sequencing results of *Gigantopelta aegis*

Sample	Bioidentifier and Body Length	Result		
		CO1	16S	28S
S1	67 (6.02mm), 71 (5.02mm)	67, —	67, 71	—, —
S2	72 (6.03mm), 82 (5.72mm)	72, —	72, 82	—, —
S3	92 (9.40mm), 109 (9.05mm)	—, —	92, 109	—, —
L1	3 (17.00mm)	—	3	3
L2	6 (17.00mm)	6	6	6
L3	5 (26.80mm)	5	5	5
L4	7 (17.00mm)	7	7	7
L5	8 (28.00mm)	8	8	8
L6	14 (34.00mm)	14	14	14

Table S3 Similarity percentage analysis of phylum-level contributions to differences between small group and large group (Cumulative contribution up to 80%)

Phylum	Average Contribution	Standard Deviation	Ratio	Cumulative Contribution	*P*-Value
Proteobacteria	0.11	0.07	1.62	0.45	0.611
Bacteroidetes	0.08	0.05	1.52	0.79	0.478
Actinobacteria	0.02	0.02	1.13	0.89	0.764

Table S4 Similarity percentage analysis of genus-level contributions to differences between small group and large group (Cumulative contribution up to 80%)

Genus	Average Contribution	Standard Deviation	Ratio	Cumulative Contribution	*P*-Value
Thiogranum	0.17	0.18	0.93	0.24	0.973
Sediminibacterium	0.12	0.04	3.44	0.41	0.001
Burkholderia	0.10	0.14	0.70	0.55	0.626
Sulfurovum	0.07	0.04	1.60	0.65	0.384
Mycobacterium	0.03	0.02	2.01	0.69	0.001
Methylovirgula	0.03	0.02	1.61	0.73	0.089
Bradyrhizobium	0.02	0.02	1.38	0.77	0.245
Pseudomonas	0.02	0.02	0.82	0.80	0.105
Chitinophaga	0.02	0.02	1.04	0.82	0.201

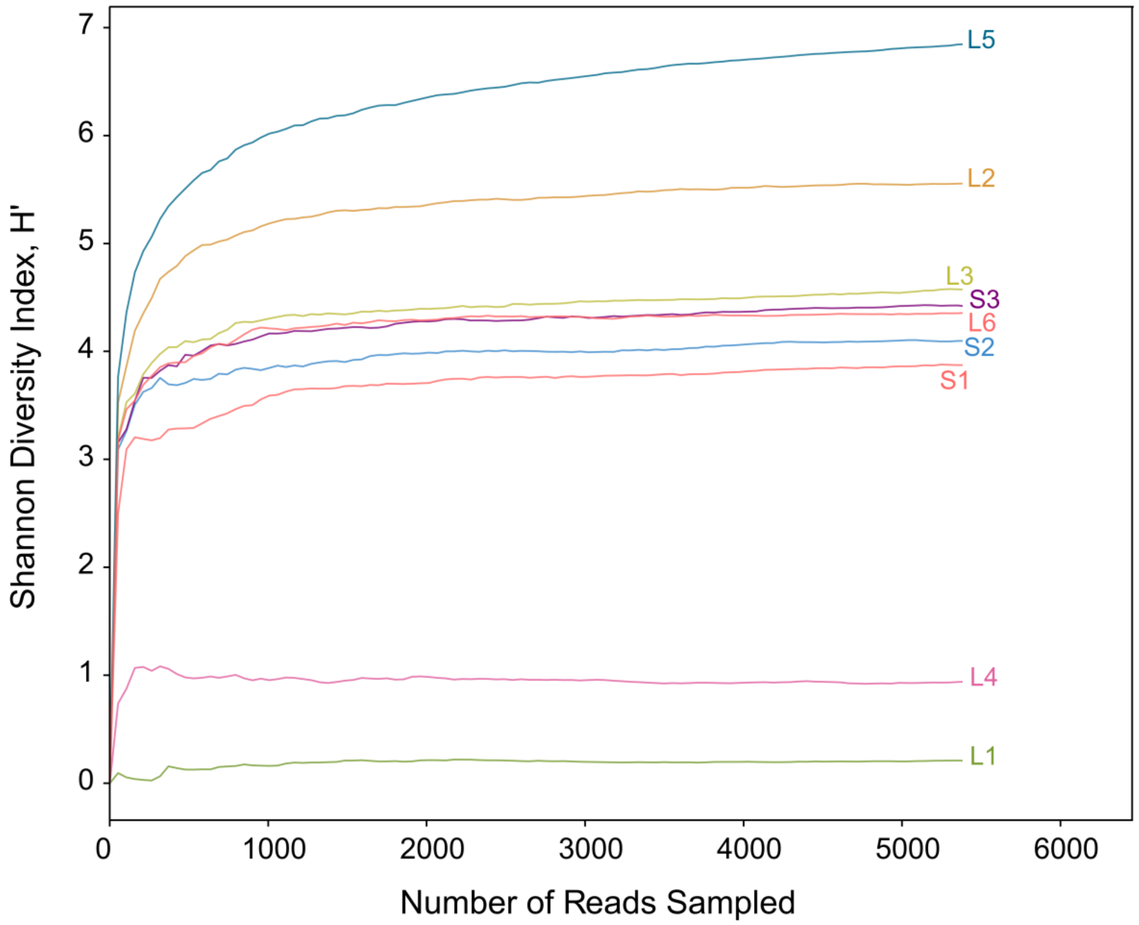

Fig. S1 Rarefaction curves of sequenced microbial samples under OTU level indicated by Shannon index. Microbial diversity in each sample has reached saturation, sequencing depth is sufficiently reliable. Samples are distinguished by their unique colors.

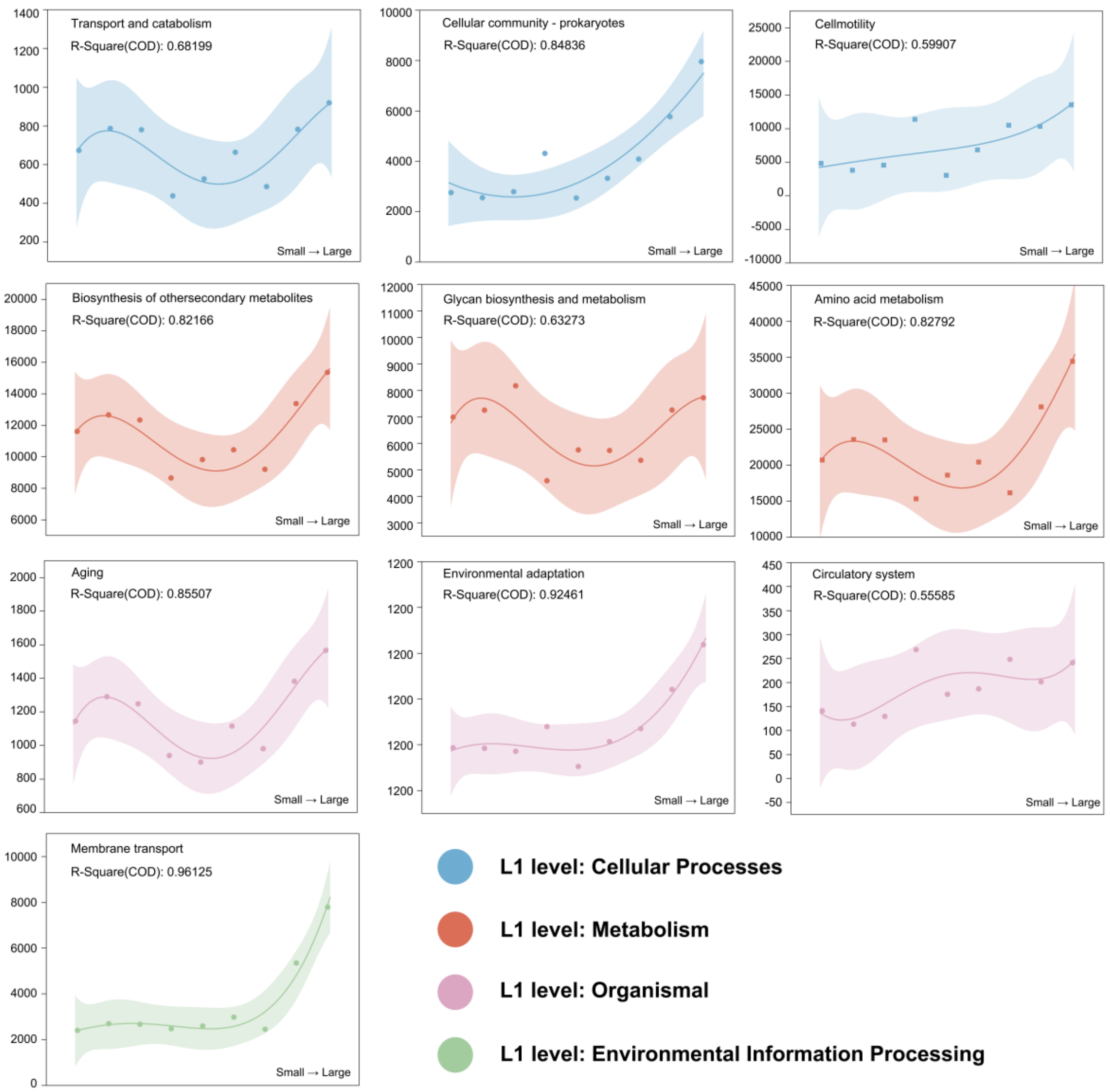

Fig. S2 Fitted curve of significantly different KEGG pathways among *Gigantopelta aegis* samples. Polynomial degree: 4; confidence interval: 95%.

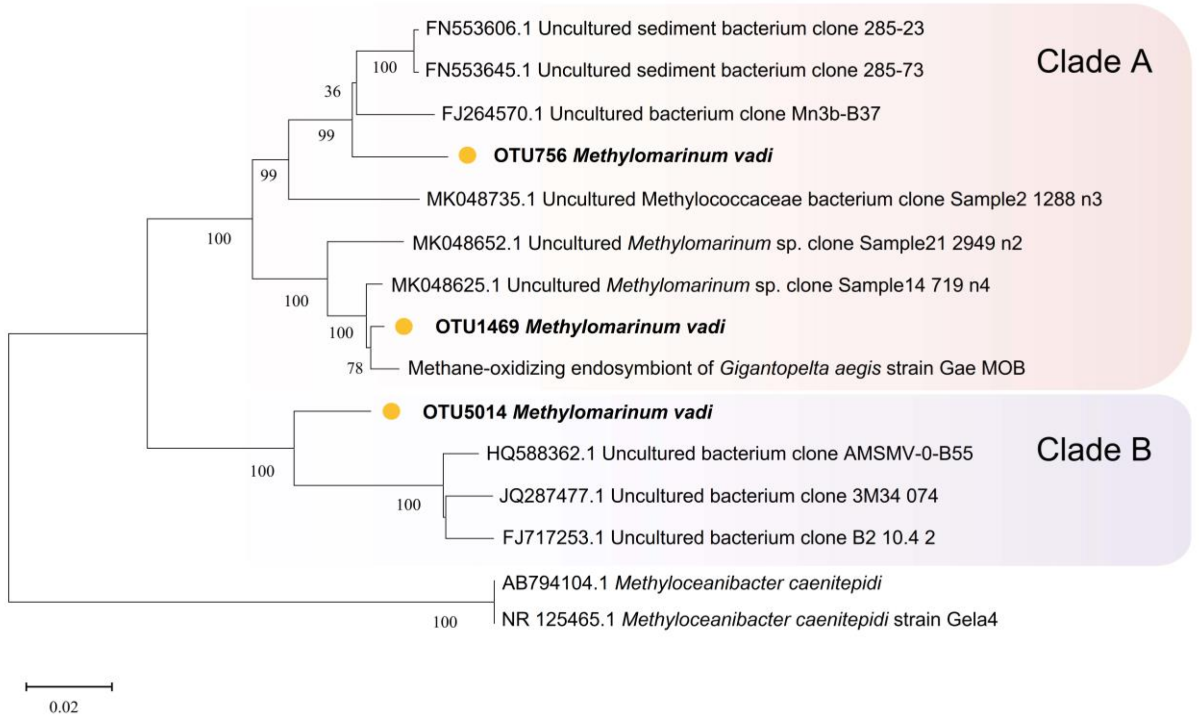

Fig. S 3 A phylogenetic tree of methane-oxidizing bacteria (MOB) *Methylomarinum vadi* and its closest relatives based on 16S rRNA gene. The bootstrap values (>50%) of relevant nodes are shown based on 1,000 replicates. The number on the branch are the result of the neighbor-joining (NJ) algorithm. Sequences from this study are shown in bold. The identified *Gigantopelta aegis* MOB is included in the tree.

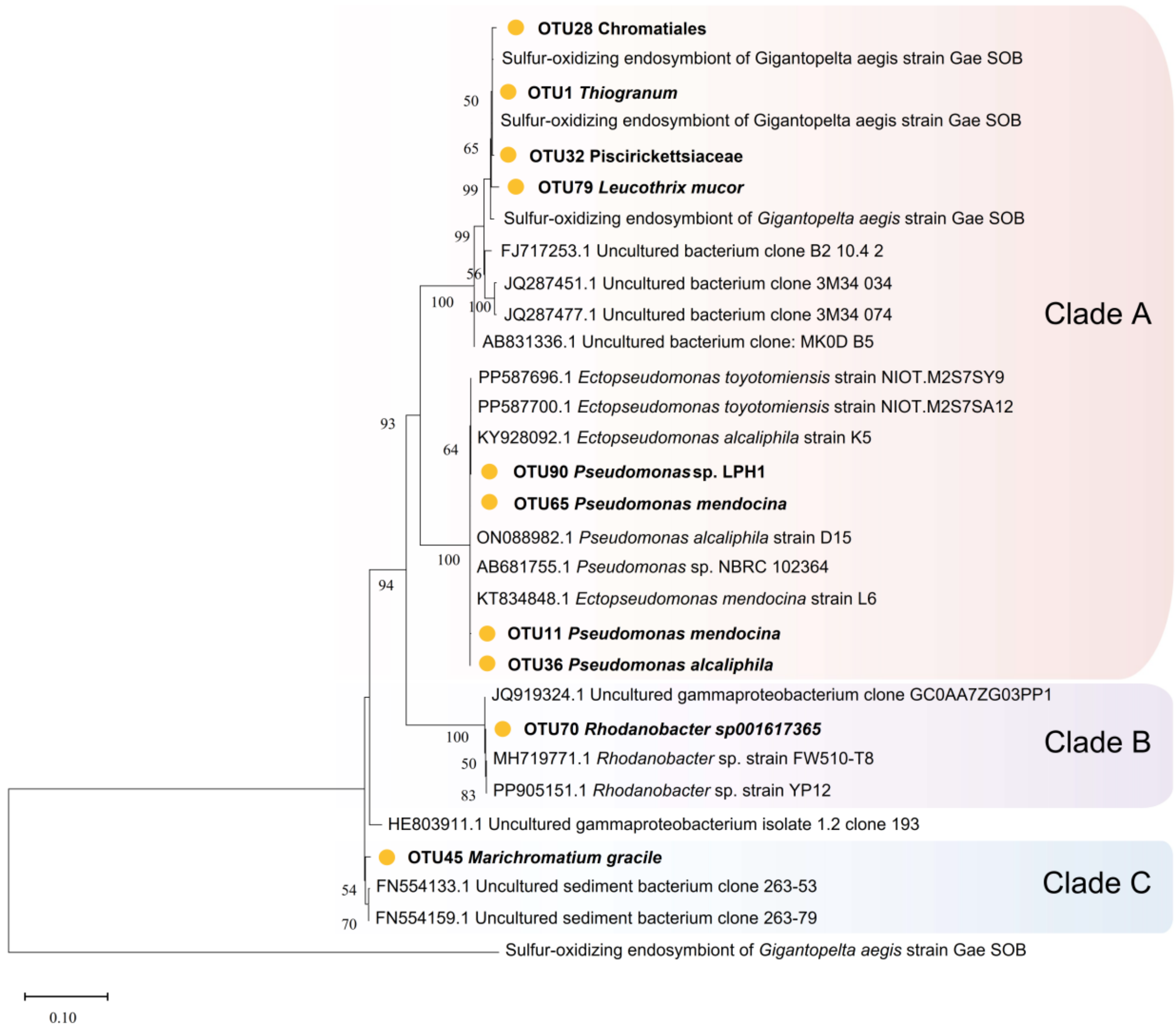

Fig. S4 A phylogenetic tree of gamaprotebacterium (top 10) and its closest relatives based on 16S rRNA gene. The bootstrap values (>50%) of relevant nodes are shown based on 1,000 replicates. The number on the branch are the result of the neighbor-joining (NJ) algorithm. Sequences from this study are shown in bold. The identified *Gigantopelta aegis* sulfur-oxidizing bacteria are included in the tree.
